# Protocol for a randomised controlled trial investigating self-help email messages for sub-threshold depression: the Mood Memos study

**DOI:** 10.1186/1745-6215-12-11

**Published:** 2011-01-13

**Authors:** Amy J Morgan, Anthony F Jorm, Andrew J Mackinnon

**Affiliations:** 1 Orygen Youth Health Research Centre, Centre for Youth Mental Health, University of Melbourne, Victoria, Australia

## Abstract

**Background:**

Sub-threshold depression is common, impairs functioning, and increases the risk of developing major depression. Although psychological treatments have been investigated for sub-threshold depression, they are costly. A less costly alternative could be an educational health promotion campaign about effective self-help for depression symptoms. The aim of the study is to test the efficacy of a low-cost email-based mental health promotion campaign in changing self-help behaviour and preventing more severe depression in adults with sub-threshold depression.

**Methods/Design:**

The project is a randomised controlled trial of an automated preventive email-intervention aimed at people with sub-threshold depression. Adults aged 18+ with sub-threshold depression (as measured with the Patient Health Questionnaire-9), who are not already receiving professional treatment for depression, are eligible for admission to the study. Internet users will sign up via the study website http://www.moodmemos.com and be randomly allocated to receive emails twice weekly for six weeks containing either self-help coping advice or general information about depression as a control. Outcomes will be assessed at the start, midpoint, and end of the intervention, as well as six months later. Outcomes assessed include symptoms, incidence of major depression, psychological distress, social and occupational functioning, coping strategies, and coping self-efficacy. The primary hypothesis is that the Mood Memo emails containing coping strategies will reduce depression symptoms and be better at preventing major depression than the control emails that contain general information about depression.

**Discussion:**

Promotion of actions an individual can take to prevent physical disease is a technique often used in public health. This study applies this approach to mental health, and explores whether a low-cost, easily disseminated email-based campaign can improve self-help coping behaviour and prevent depression in adults with sub-threshold depression.

**Trial Registration:**

Australia and New Zealand Clinical Trials Register (ANZCTR): ACTRN12609000925246

## Background

Evidence suggests that depressive disorders exist on a continuum, rather than as qualitatively distinct syndromes [1]. Depressive symptoms that fall short of diagnostic criteria (variously termed sub-threshold, sub-clinical, sub-syndromal, mild, or minor depression) are prevalent [2], cause significant functional impairment [1,3], have considerable economic costs [4], and increase the risk of developing major depressive disorder [5]. Sub-threshold depression is of major public health significance because of its substantial population disease burden which, in turn, arises from its high prevalence combined with significant disability to the individual.

Treatment of sub-threshold depression with psychological interventions (e.g., cognitive behaviour therapy) is moderately effective [6]. However, addressing the problem in formal health-care systems may not be an ideal solution, as health care resources are already stretched treating those with depressive disorders, yet there remain many with depressive disorders who do not receive treatment [7]. An alternative approach is self-help that can be used by individuals without the need for professional guidance. Self-help strategies, such as exercise or taking time off work, are liked by the public and are often used by those with depressive symptoms [8]. However, some commonly used strategies may be ineffective or even harmful (e.g., drinking alcohol). [9].

Our previous research has identified a set of self-help coping strategies judged to be effective by an international group of depression experts. These strategies include becoming more physically active, improving sleep habits, and doing activities that give a sense of achievement [10]. Health promotion campaigns encouraging the public to use effective self-help strategies for depression symptoms could be a cost-effective solution to reducing population disease burden [11]. This would be analogous to physical health promotional campaigns advocating behaviour change to reduce risk of disease (e.g., heart disease). This approach is rarely used in the mental health field. It could be applied to individuals with sub-threshold depression, with the aim of reducing symptoms and also to reduce the risk for the development of major depression. Intervening early could also improve functioning and prevent progression to other undesirable outcomes such as harmful substance use.

The internet is an excellent medium for the promotion of self-help messages, as it has significant population penetration, can cost-effectively reach large numbers of people, and is a frequently-used source of information about mental health problems [12]. In contrast to media traditionally used to promote health messages, internet technology potentially affords greater personalisation and interactivity, which may lead to greater persuasion and increase the likelihood that an individual will change their coping strategies [13]. Furthermore, website-based interventions for depression have proved effective [14], and health messages sent via email have improved health behaviours, such as improving diet and physical activity levels [15].

### Aim

The aim of the study is to determine the effectiveness of a mental health promotion campaign encouraging the use of a range of self-help coping strategies for sub-threshold depression, which have been identified by experts in the field. The study will evaluate the feasibility of the internet as a means of intervention, and will evaluate the impact of the intervention on depression symptom severity and incidence rates of major depression.

## Methods/Design

### Design

The project is a randomised controlled trial of an automated preventive intervention for adult internet users with elevated levels of depressive symptoms that do not meet diagnostic criteria for major depression (sub-threshold depression). The period of intervention is 6 weeks. Assessments are undertaken at baseline, 3-weeks, 6-weeks, and at 6-months after the end of the end of the intervention. The study was approved by the University of Melbourne Human Research Ethics Committee (HREC 0931313), and is registered with the Australian New Zealand Clinical Trials Registry (ACTRN12609000925246).

### Participants

Participants will be admitted to the trial if they are aged 18 or over; have sub-threshold symptoms of depression; are not receiving treatment for depression from a professional (e.g., doctor, counsellor, psychologist), not including maintenance antidepressant medication for 6 months or more; are a resident of Australia, New Zealand, UK, Ireland, Canada or the USA; and have access to the internet at least weekly. Sub-threshold symptoms are defined as two to four symptoms of depression [16] experienced most of the time for two or more weeks, which have affected work, home, or social functioning. Participants will be recruited to the project website http://www.moodmemos.com from internet-based sources, such as paid advertising on Google, Yahoo, and Facebook, and promotion through websites, forums, blogs, and email groups related to depression, mental health, research, or depression risk-factors. It is planned that the project website homepage will be archived at the end of the trial using a service such as WebCite[17].

### Sample size

The effect size of the intervention is anticipated to be small, given that indicated prevention of depression has a small effect size of d = 0.23 [18]. Similarly, unsupported internet-based treatment of depression has a small effect size of d = 0.25 [14]. A power analysis indicated that a sample of 393 per condition would give 80% power to detect a small effect size (0.2 standard deviations between conditions) on a continuous outcome measure assuming a correlation of 0.5 between pre- and post-test scores [19]. Because depression internet trials have a median dropout rate of 33% [20], a total sample of 1200 participants will be aimed for.

### Trial procedure

The administration of the intervention is fully automated using PHP 5 and a MySQL 5 database. Participants who visit the project website and give their informed consent to participate are screened for sub-threshold depression. They will be categorised into one of three depression status groups based on their results:

1) Sub-threshold depression: 2 to 4 symptoms of depression present for more than half the days or nearly every day (except for suicidality, which is counted as a symptom if present for only several days or more), for 2 weeks or more, with significant impact on functioning

2) Probable major depression: 5 or more symptoms of depression present for more than half the days or nearly every day (except for suicidality, which is counted as a symptom if present for only several days or more), for 2 weeks or more, with significant impact on functioning

3) Non-depressed: not meeting criteria for sub-threshold or major depression, i.e. too few symptoms, too short duration, little or no impact on functioning.

Depression symptoms are defined as the nine symptoms in criterion A of DSM-IV Major Depressive Episode [16] assessed with the PHQ-9 (see Assessment below). Depression status is scored by the system and trial eligibility is determined immediately, based on admission criteria. Those whose results indicate major depression or non-depressed will be given feedback on their score and informed that they are not eligible to participate. In addition, those with major depression will be encouraged to seek professional help and will be directed to a section on the website that provides contact details of organisations that provide help or support for mental disorders, broken down by country.

Participants with a positive screen for sub-threshold depression will be assessed to see whether they meet further inclusion criteria (see admission criteria above) and asked to provide their name or a pseudonym and an email address. An email will be sent to the address provided, which will contain a link to the baseline questionnaire package. Once the baseline assessment is completed, participants will be randomised to the active group or control group using the mt_rand() PHP script. Immediately following randomisation, participants will be sent their first Mood Memos email.

Each participant will receive emails twice a week for six weeks. Emails will be automatically generated by the system and personalised with relevant information (e.g., name) from the database. One email reminder per assessment point will be sent to those participants who have not completed assessments one week after being sent the email inviting completion of assessment. Participants who do not complete assessments will continue to receive the intervention, and may rejoin assessment at later timepoints.

### Intervention

Both groups receive a set of 12 Mood Memos emails over 6 weeks. The Mood Memos emails are written in html to look as engaging as possible. Emails are also written in plain-text for participants who cannot or who choose not to receive html emails. Email design and layout was tested in all of the major email clients, using the services of Litmus [21] and Email on Acid [22]. Each email includes identification of the recipient and how they got on the mailing list, and provides the opportunity to withdraw from the study by choosing not to receive further Mood Memos emails and/or complete further assessment.

### Active group

Email messages for the intervention group are based on self-help strategies endorsed by depression experts [10] (see Table 1 for an overview). These self-help strategies were expanded into lengthier email messages. The emails aim to change participant behaviour rather than passively increase knowledge, and as such, their design was informed by theories of behaviour change, persuasion, health communication, and communication on the web [23-32]. Each message contains a brief overview of the strategy, a lengthier explanation of the strategy, why the strategy would work, tips on how the strategy could be implemented, suggested solutions for barriers to implementing the strategy, and an appeal to commit to implementing the strategy. The content of the messages (e.g., strategy rationales, potential barriers and solutions) was derived from professional books, and posts on depression forums by consumers [33-37]. Messages were refined further by a working group of mental health researchers (including consumer researchers). The messages have minimal tailoring to individual participant characteristics [38]. For example, links to internet resources are tailored based on country of residence and the wording advocating each strategy is tailored to the participant's familiarity with using each strategy [29], as indicated during the baseline questionnaire. The strategies are ordered from highest to lowest feasibility to carry out, based on rankings from the original expert study [10]. This is consistent with clinical advice [39,40] to start homework with simple, feasible tasks and build up with time and success.

**Table 1:** Overview of Mood Memos Emails

Order	Active	Control
**1**	Get out of the house	What is depression?

**2**	Fight the desire to do nothing	What types of depression are there?

**3**	Set yourself a small goal and reward yourself for reaching it	Can depression cause physical health problems?

**4**	Eat well	Depression and other mental health problems

**5**	Improve your sleep habits	What is the history of depression?

**6**	Do something you enjoy and that gives you a sense of achievement	What causes depression?

**7**	Talk to someone supportive about your problems and how you feel	Who is at risk of depression?

**8**	Get active	How common is depression?

**9**	Do what has worked for you in the past	Burden of depression

**10**	Let others know how you are feeling	Is depression a weakness?

**11**	Ask someone you trust to help you get out and do things	Does depression re-occur?

**12**	Learn relaxation techniques	Depression across cultures

### Control group

The control group will receive email messages containing general information about depressive disorders (see Table 1 for an overview). The messages are designed to be interesting but of no therapeutic value. Their role is to control for the effect of receiving emails with depression-related content. The order of the messages was chosen to follow a reasonably logical progression from basic or essential information to more advanced or less essential information. The messages are not tailored, and critically, they do not include suggestions for action or setting goals. Message content was sourced from recently published psychiatry textbooks, and supplemented with recent reviews or meta-analyses as required.

### Assessment

Assessment is conducted at baseline (immediately prior to receiving the first Mood Memos email), midway through the intervention (3 weeks post-baseline), at the end of the intervention (6 weeks post-baseline), and 6 months after the end of the intervention (see Table 2 for outcome measures and time points of assessment). All assessment is based on self-report and is completed on the project website. The assessment is aimed to take little time to complete in order to discourage dropout attrition [41] whilst providing critical data. Time to complete questionnaires is estimated to range from 2 minutes (mid-intervention and 6-month follow-up assessment) to 6 minutes (for baseline and post-intervention assessment). The primary outcome is depression symptom severity (assessed with the PHQ-9 [42]). This outcome will also be categorised to yield probable depression diagnoses. Secondary outcomes are psychological distress (assessed with the K10 [43]) and level of functioning (assessed with the Work and Social Adjustment Scale [44]).

**Table 2:** Outcome measures and timepoints of assessment

Assessment	Screening	Baseline	Mid-intervention	Post-intervention	6 months follow up
List of Threatening Experiences		X			

Sociodemographics		X			

PHQ-9	X		X	X	X

K10		X	X	X	X

Work and Social Adjustment Scale		X	X	X	X

Self-efficacy		X		X	

Knowledge of depression		X		X	

Coping questionnaire		X		X	

Help-seeking question			X	X	X

Qualitative feedback				X	

### Depression

The depression module of the Patient Health Questionnaire (PHQ-9) [45] will be used to assess depression. It is a self-rated questionnaire that makes probable diagnoses of mental disorders based on DSM-IV criteria [46]. The PHQ-9 consists of items covering the 9 symptoms of criterion A for a DSM-IV diagnosis of Major Depressive Episode [16]. Each item is rated in terms of its frequency over the last two weeks, from 0 (not at all) to 3 (nearly every day). Its structure means it can be used both to make probable diagnoses of major depression and sub-threshold depression, as well as to measure the severity of symptoms (0 to 27). An additional question asks about the impact of the symptoms on the person's functioning, analogous to criterion C of DSM-IV Major Depressive Episode [16]. The PHQ-9 has excellent reliability assessed by internal-consistency [45,47,48], adequate test-retest reliability [45,49], excellent criterion validity [50], and is sensitive to change [47,51]. It has been used in studies of internet treatment of depression [52], to assess rates of sub-threshold depression in the community [53], and to measure treatment of sub-threshold depression in primary care [54].

### Psychological distress

The K10 [43,55] will assess non-specific psychological distress. The K10 measures 10 symptoms of mental health in the anxiety-depression spectrum, using a 5-point response scale based on the amount of time the respondent has experienced the symptom in the past 30 days. Using the Australian scoring method [56] scores range from 10 (no distress) to 50 (severe distress). It is widely used as a screening tool in population surveys [56,57] and as an outcome measure in mental health services [58], as well as in some depression treatment studies [52,59]. It has good internal consistency [55,60] and is a good discriminator of community cases and non-cases of DSM-IV disorders [61]. It also has good reliability and validity when completed on the internet [60].

### Functioning

Functioning will be assessed with the Work and Social Adjustment Scale (WSAS) [44]. This is a brief, 5-item scale that measures impairment attributable to an identified problem in work, home management, social activities, private leisure activities, and ability to form and maintain relationships. Scores range from 0 to 40, with a score above 20 suggesting moderate to severe psychopathology, scores between 10 and 20 suggesting significant functional impairment but less severe symptomatology, and scores below 10 being associated with subclinical populations. The scale has strong psychometric properties, with good internal consistency, test-retest reliability, alternate forms reliability, and good convergence with scores on the Hamilton Rating Scale for Depression and patient ratings of perceived improvement [44]. It has also been used in online studies of depression treatment [62,63].

Several other assessment tools will be used in addition to those described above.

### List of Threatening Experiences

The List of Threatening Experiences (LTE) is a brief measure of the occurrence of negative life events in the past six months. It records common events that are likely to be threatening and of aetiological importance to psychiatric disorders [64,65]. Twelve events (e.g., you broke off a steady relationship) are scored as being present or absent in the past six months. This measure was included at baseline as a potential predictor of intervention response, as it could be argued that the self-help strategies endorsed in the Mood Memos would not be helpful for those whose depression symptoms were due to significant life events. A reliability study showed the LTE has adequate test-retest reliability, agreement ratings with other informants on the events were good, and it has good concurrent validity [65]. The scale has been used in several large population surveys (e.g. [66,67]).

### Coping questionnaire

Participants are asked to rate the frequency and perceived helpfulness of 26 coping strategies for depression symptoms over the past month at baseline and at post intervention. The coping strategies include the 14 used in the Mood Memos emails, as well as other strategies that were rated in the expert study [10] as relatively unhelpful (e.g., *you drank alcohol*) or unlikely to be helpful (e.g., *you had a warm bath*). These were assessed to determine participants' coping repertoire at baseline, whether the intervention changed their coping strategies, and whether this was related to intervention response. Frequency of use is rated on a 5-category scale (*not at all*, *infrequently*, *moderately frequently*, *very frequently*, *don't know*), and perceived helpfulness on a 5-category scale (*not at all helpful*, *a little helpful*, *moderately helpful*, *very helpful*, *don't know*). Participants are asked to rate a strategy's perceived helpfulness only if they have indicated they had used the strategy (*infrequently *to *very frequently*) in the past month.

### Knowledge of depression

A 12-item questionnaire was designed to measure knowledge of depression. This questionnaire will be used to add credibility to the control intervention and as a proxy measure for whether the control emails are read. It will be administered at baseline and at post intervention. Each question relates to the content of one control Mood Memos email. The response scale is *true*, *false*, *don't know*, with one point for each correct answer, giving a range of scores from 0 to 12. The questionnaire was piloted on the working group, and six members of the public.

### Self-efficacy

Participants will be asked "How confident are you in your ability to help yourself improve your depression symptoms?" at baseline and at post intervention. The response scale used is *not at all confident*, *a little confident*, *moderately confident*, *very confident*, *don't know*. Self-efficacy was measured as an intermediary effect because it is a predictor of behaviour change [30] and short-term health promotion campaigns often only demonstrate improvement in intermediary effects rather than behaviour change.

### Other

Characteristics of participants including age, gender, personal history of depression or psychotic disorders, education level, and ethnic background will be assessed at baseline. These are collected as potential predictors of intervention response. Participants will also be asked at each assessment point whether they had sought help from a health professional recently for their depression symptoms, to eliminate this as a confounding variable. Qualitative data on participants' experience of the study will be collected via a feedback form at the post-intervention assessment.

### Statistical analysis

Primary effectiveness analyses will be undertaken on an intention-to-treat basis. Any participants who were randomised but who withdraw from the study, or who take up alternative treatments, will be included in the analysis as randomised. Continuous outcomes (PHQ-9 scores, K10 scores, WSAS scores) will be evaluated using mixed models for repeated measures (MMRM) [68]. This method of analysis will be utilised because of its ability to include participants with missing data under the assumption that observations are missing at random (MAR). The inclusion of an additional measurement occasion halfway through the intervention should increase the tenability of the MAR assumption. *A priori *contrasts will compare change from baseline at the primary endpoint (post-intervention) and at 6-months follow up. Dichotomous outcomes will be defined. These include the development of probable major depression (as indicated by PHQ-9 results) in participants who did not meet this criterion at baseline and remission from sub-threshold depression in those who fell into this category at baseline. Relative risk will be calculated and tested for significance. The numbers needed to treat to avoid one person developing major depression and to achieve one remission from sub-threshold depression will be calculated.

In addition to intervention group, a number of variables will be examined to see if they predict outcomes: age, gender, education level, past diagnosis of depression, past diagnosis of psychotic disorder, whether they sought help during the intervention, baseline self-help for depression self-efficacy, and whether they had experienced an adverse life event in the six months prior to the development of sub-threshold depression.

Other outcomes include coping strategies used, knowledge of depression, and self-help for depression self-efficacy. In order to determine whether any change in depression might be due to changes in coping behaviour, the coping strategies used by participants will be evaluated. Usage rates and change in usage of each strategy in each intervention group will be compared pre- and post-intervention. Change in use of each strategy will be evaluated using logistic regression expressed as odds ratios with their 95% confidence intervals. Each strategy's mean perceived helpfulness for participants using them will also be compared pre- and post-intervention between groups.

Assessment attrition will be described at each assessment point. Participant characteristics and levels of depression will be analysed as predictors of missingness within each group. Email usage adherence will be explored by comparing frequencies of email image downloads between groups. Possible multiple enrolments will be explored by examining small variations and patterns in participant names, IP addresses and email addresses [69].

## Discussion

This study is part of a wave of new internet interventions to manage disease and improve health behaviour. Public health interventions disseminated via the internet have the potential for great impact by reaching large populations and providing an interactive and tailored intervention. As people from all over the world can be recruited, internet-based trials have the potential for large sample sizes and improved external validity [70]. Although initial set-up costs may be relatively high, the scalability of internet interventions means lower overall costs for large samples, particularly if data collection is completely internet-based. Automating data collection and outcome measurement also minimises the potential for biases introduced by human observers, and ensures the uniformity of intervention delivery [70]. The study's unique strengths are its promotion of self-help strategies endorsed by expert consensus and its use of messages designed with input from theories of behaviour change and persuasion. Another strength of the study is the use of a content-relevant attention placebo control condition, rather than a waitlist or no-contact control group, as is often used in depression prevention research [18]. This eliminates the possibility of placebo effects accounting for any intervention effects found.

Automating study processes and setting the intervention on the internet has a number of advantages, but also presents several challenges. Internet interventions frequently demonstrate high non-usage attrition and high dropout attrition [41] due to less participant supervision and ease of discontinuing. Dealing with attrition and missing data is a fundamental methodological challenge. Fully automated online interventions can be subject to multiple enrolments, which may bias randomisation [71], however this is less of a problem in studies that do not provide financial incentives for participation. In addition, there are strategies to detect multiple enrolments, such as analysing similar or identical IP addresses and usernames [69]. Unequal access to the internet in the community is frequently cited as a criticism and challenge to external validity [72]. However, the study is a first test of the value of email-based self-help messages for depression, and is not proposed as the only solution, nor a solution that will benefit everyone in the community.

Conducting assessments of outcomes on the web also poses several limitations. There are concerns about the equivalence in validity and reliability of web-based assessments to paper-and-pencil based assessments [73], though when this has been evaluated with depression or mental health assessments there is little evidence of differences between the two administration modes [74,75]. In addition, all outcome measures are self-rated by definition, rather than the preferred method of clinical ratings or diagnostic interviews. Self-rated assessment may be vulnerable to respondent bias [76] and other causes of depression (e.g., medication side effects, physical diseases) cannot be ruled out as would occur in a diagnostic interview, nor are co-morbid mental disorders assessed. However, the study asks participants at baseline about bereavement and history of mania or psychotic disorders. The presence of these could be used in a sensitivity analysis. We decided not to exclude individuals with these profiles from participating, because we wanted to test whether the approach is feasible and effective as a first step, so we kept exclusion criteria to a minimum.

Limited research suggests that email-based interventions are a promising method of delivering health behaviour change interventions. In contrast to website-based interventions, email-based interventions may be more convenient to participants because they do not have to take conscious action in order to receive content. These interventions have the advantage of *stickiness *[77,78], the concept that ideas and intentions are more likely to 'stick' (i.e. make an impact and be remembered) when they appear frequently in the person's environment. They are also potentially easier to implement than complex website-based interventions and hence could have better cost-effectiveness. Website-based depression interventions can be effective but require considerable user commitment and usually health professional involvement [79]. If the email-based approach used in this study proves effective, the Mood Memos intervention could be broadly disseminated and be a low-cost way of reducing the population burden of depressive symptoms.

## Competing interests

The authors declare that they have no competing interests.

## Authors' contributions

The study is part of the doctoral dissertation of AMo, who is principal investigator. AMo participated in the study design, technical development, participant recruitment, trial co-ordination and drafted the manuscript. AMo is supervised by AJ and AMa, who both participated in the study design. All authors have read and approved the final manuscript.
